# Characterization of biomechanical properties of cells through dielectrophoresis-based cell stretching and actin cytoskeleton modeling

**DOI:** 10.1186/s12938-017-0329-8

**Published:** 2017-04-04

**Authors:** Guohua Bai, Ying Li, Henry K. Chu, Kaiqun Wang, Qiulin Tan, Jijun Xiong, Dong Sun

**Affiliations:** 1 Key Laboratory of Instrumentation Science and Dynamic Measurement, Ministry of Education, North University of China, Room 418, Building No. 14, No. 3 Xueyuan Road, Taiyuan, 030051 Shanxi China; 2 Department of Mechanical and Biomedical Engineering, City University of Hong Kong, 83 Tat Chee Avenue, Kowloon Tong, Hong Kong, SAR of China; 3 Department of Mechanical Engineering, The Hong Kong Polytechnic University, Hung Hom, Kowloon, Hong Kong, SAR of China; 4 Department of Biomedical Engineering, College of Mechanics, Taiyuan University of Technology, No. 79, West Yingze Street, Taiyuan, 030024 Shanxi China

**Keywords:** Dielectrophoresis, Cell stretching, Cytoskeleton model, Optical tweezers, Mechanical property

## Abstract

**Background:**

Cytoskeleton is a highly dynamic network that helps to maintain the rigidity of a cell, and the mechanical properties of a cell are closely related to many cellular functions. This paper presents a new method to probe and characterize cell mechanical properties through dielectrophoresis (DEP)-based cell stretching manipulation and actin cytoskeleton modeling.

**Methods:**

Leukemia NB4 cells were used as cell line, and changes in their biological properties were examined after chemotherapy treatment with doxorubicin (DOX). DEP-integrated microfluidic chip was utilized as a low-cost and efficient tool to study the deformability of cells. DEP forces used in cell stretching were first evaluated through computer simulation, and the results were compared with modeling equations and with the results of optical stretching (OT) experiments. Structural parameters were then extracted by fitting the experimental data into the actin cytoskeleton model, and the underlying mechanical properties of the cells were subsequently characterized.

**Results:**

The DEP forces generated under different voltage inputs were calculated and the results from different approaches demonstrate good approximations to the force estimation. Both DEP and OT stretching experiments confirmed that DOX-treated NB4 cells were stiffer than the untreated cells. The structural parameters extracted from the model and the confocal images indicated significant change in actin network after DOX treatment.

**Conclusion:**

The proposed DEP method combined with actin cytoskeleton modeling is a simple engineering tool to characterize the mechanical properties of cells.

## Background

The mechanical properties of cells are closely related to a number of biological functions, such as cell differentiation, aging, motility, metastasis, and mechanotransduction [1–4]. Several studies have recently suggested that cellular properties can be utilized as label-free biomarkers for disease diagnosis [5–7]. For instance, metastatic cancer cells are softer than normal cells [6]. Red blood cells (RBCs) from patients with sickle cell trait display lower deformability than healthy RBCs [7]. Hence, the effectiveness of a certain chemotherapy drug or medicine on cells can be evaluated by quantitatively comparing the deformation behavior of cells after treatment, and this approach brings new insights into the pathogenesis and treatment of various diseases [8, 9].

Over the past several decades, numerous engineering techniques, including atomic force microscopy (AFM), micropipette aspiration, magnetic tweezing, optical tweezing, optical stretching, hydrodynamic stretching, deformability cytometry, and electroporation deformation, were developed for cell deformability test [10–17]. The AFM approach and optical approach are widely adopted owing to their reliability and accuracy in obtaining data through the use of a sophisticated system. These two methods, however, suffer from low throughput and lengthy system setup. Dielectrophoresis (DEP) has increasingly received attention in recent years because it can be used as a simple alternative to other conventional approaches in characterizing the mechanical properties of a cell [18–22]. DEP-based cell electro-deformation was first reported in 1984 [23], and this technique involves the basic working principle of polarizing and stretching cells by using a non-uniform electric field produced by a pair of electrodes. The main advantage of this method is that a relatively large stretching force (e.g., nanoNewton) can be applied on cells, and multiple cells can be stretched simultaneously by a pair of electrodes. Nevertheless, the use of a strong electric field to produce a stretching force may lead to cell lysis caused by Joule heating [24] and structural rearrangement in the membrane [25].

The mechanical behavior or deformation of a stretched cell is largely determined by the cytoskeleton network, which comprises actin filaments, microtubules, and intermediate filaments [14, 26]. Several studies have reported that cell deformation is dominantly associated with actin filaments [27, 28], and actin filaments were confirmed to be influenced by actin concentration, density of cross-links, and prestress [26, 29]. To date, a number of models have been developed and used to describe the rheological properties and mechanical behaviors of cytoskeleton; these models can be categorized as continuum-, structure-, and polymer-based models [28, 30]. Continuum-based models assume a cell as a homogeneous and continuous medium when analyzing stress–strain relationship. These models can be further classified as linear elastic [31], hyperelastic [32], and viscoelastic models [33]. Structure-based models utilize discrete structural elements, which commonly include tensegrity [34], 3D random network [35], and spring network [28], to represent the microstructural component of a cell. Polymer-based models utilize the structure of actin filaments or the morphology of polymer networks as elements to predict cytoskeleton properties. Mikado and MacKintosh-derived worm-like chain (WLC) models are two typical examples of polymer-based models.

In our early work [26], a MacKintosh-derived WLC-based actin microstructural model was developed and this model utilized actin filaments (F-actin) and actin-binding proteins (ABPs) as basic elements to represent the cytoskeleton network. Structural parameters of the model could be estimated by fitting experimental data obtained from the optical cell stretching experiments, allowing further quantitative analysis on the biomechanical behavior. In the present study, we extend our previous work by fitting the experimental results of DEP-based stretching into an actin microstructural model to probe the mechanical properties of cells. NB4 cells from a leukemia cell line were examined, and their biological properties were altered by treatment with the chemotherapy drug doxorubicin (DOX). Studies such as [36] have demonstrated that DOX can induce apoptosis in leukemia cells, resulting in alterations in the actin cytoskeleton structure. A microfluidic chip was adopted as the cell manipulation tool to apply a DEP force on a cell to achieve single-cell stretching. First, we examined different approaches used to estimate the DEP force applied via a microfluidic chip. We performed another set of stretching experiments with the same cell type by using optical tweezers (OTs), and then we compared the applied stretching forces and cell deformation behaviors under these two methods. Structural parameters of the cytoskeleton model could then be extracted by fitting the stretching experimental result into the model [26] to probe the biomechanical properties of cells after drug treatment. We concluded that the proposed DEP method combined with actin cytoskeleton modeling is a simple and cost-effective manipulation tool to characterize cells, and this method is an important alternative to the existing OT-based stretching method.

## Methods

### Cells and media

NB4 cells from a leukemia cell line were cultured in RPMI-1640 medium (Invitrogen) supplemented with 10% fetal bovine serum (Gibco BRL) and 100 U/mL of penicillin–streptomycin (Sigma-Aldrich). DOX (Sigma-Aldrich) was used to alter the biological properties of NB4 cells, and NB4-DOX cells were cultured in 0.05 μM DOX medium for 96 h. All cells were cultured in a humidified incubator with 5% CO_2_ at 37 °C. To conduct DEP-based cell stretching experiments, the cells were resuspended in an isotonic buffer medium consisting of 8.5% sucrose, 0.3% dextrose, and 20 mg/L CaCl_2_ [37]. For confocal fluorescence imaging, the cells were fixed with 4% paraformaldehyde and permeabilized with 0.5% Triton X-100 for 10 min at room temperature; afterward, the actin cytoskeleton of NB4 cells was stained with rhodamine-phalloidin (Invitrogen) for 10 min.

### Experimental setup for DEP cell stretching

**Fig. 1:**
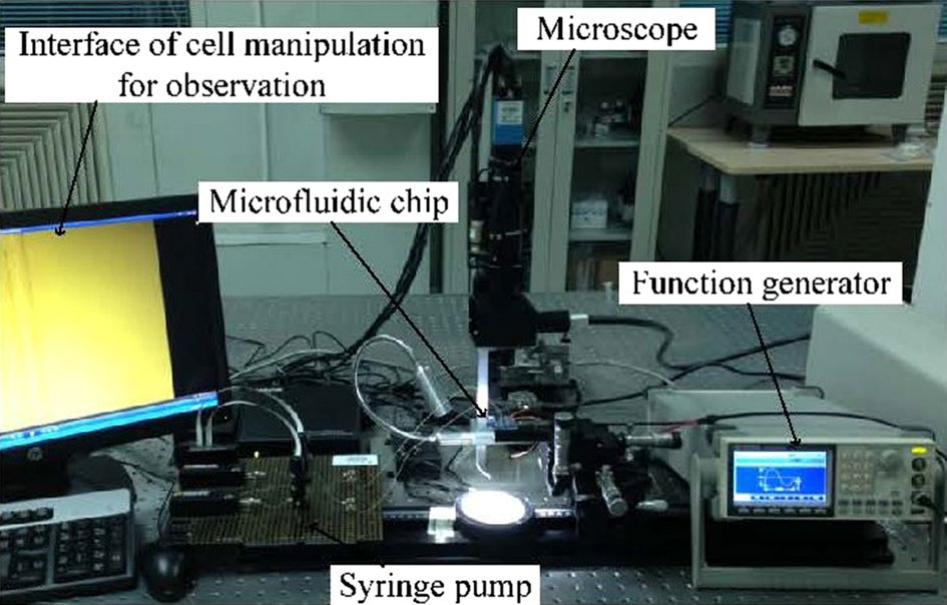
Experimental platform for dielectrophoresis-based cell stretching

**Fig. 2:**
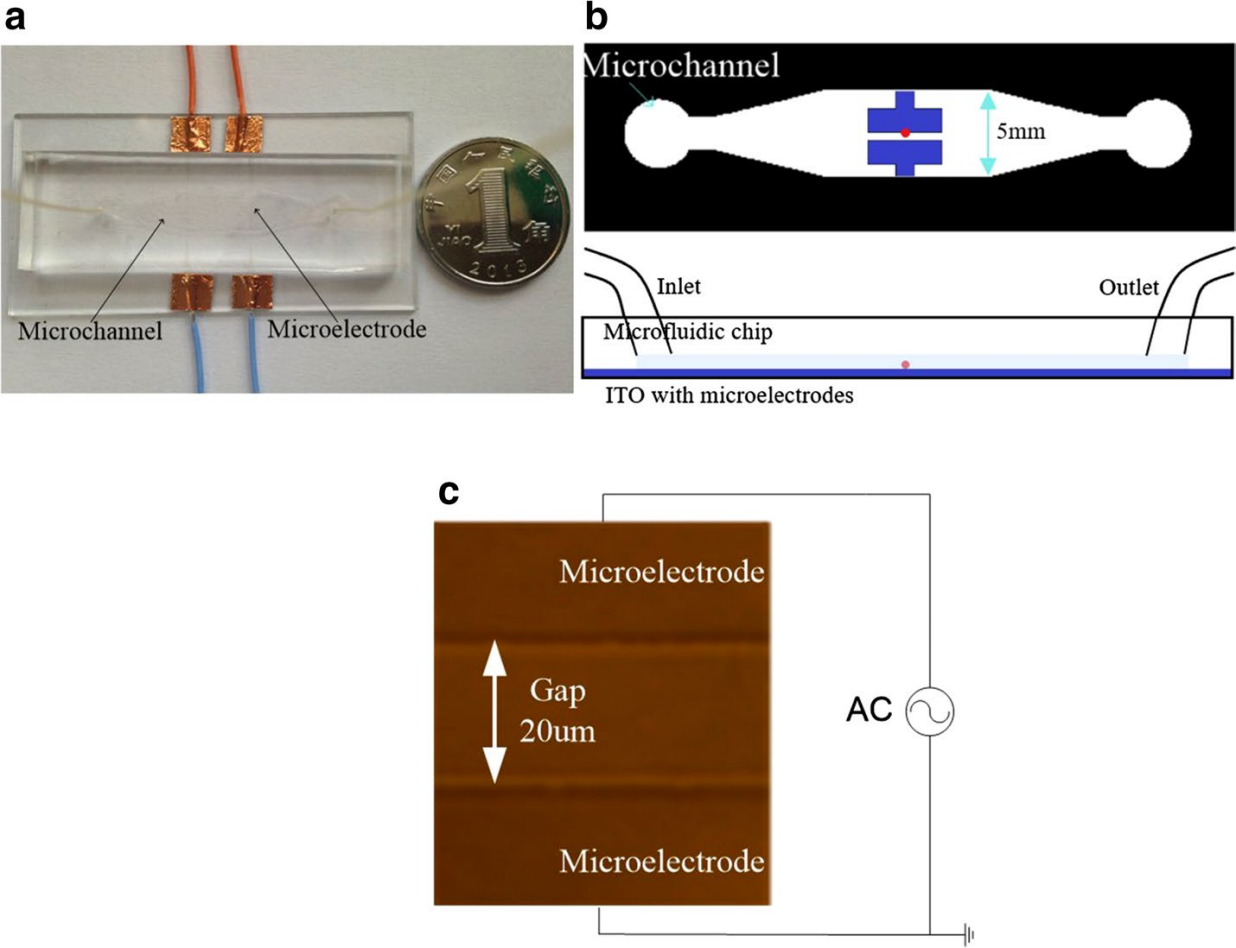
Microfluidic chip design. **a** Image of the chip; **b** schematic diagram showing the microelectrodes and the microchannel in the microfluidic chip; and **c** image captured under the microscope

An experimental platform consisting of a function generator, a positioning table, an optical microscope unit, a syringe pump, and a computer system was set up for DEP-based cell stretching and manipulation experiments (Fig. 1). A microfluidic chip with integrated microelectrodes was placed on the positioning table for viewing under the optical microscope. Figure 2a, b show the design of the microfluidic chip. The microchannel has overall dimensions of 5000 μm by 50 μm, and cell containing medium was injected into the channel by the syringe pump. The integrated microelectrodes with a gap width of 20 μm (Fig. 2c) were patterned on an indium tin oxide (ITO)-coated glass slide by using photolithography. Details of the chip fabrication was presented in our earlier work [8].

To stretch the cells, the microfluidic chip was connected to a function generator to produce a non-uniform electric field in the microenvironment via a pair of electrodes. The frequency of the electric field was selected so that a net DEP force was applied on the cells to lead them toward the electric field maxima. Based on this principle, the cells were manipulated and captured by one of the two electrodes. Given that the conductivity of the medium *σ*_*m*_ is lower than that of the cell cytoplasm *σ*_*c*_ (i.e., *σ*_*m*_ < *σ*_*c*_), the cells started to stretch and elongate along the electric field lines [38]. Finite element software COMSOL Multiphysics was used to simulate the distribution of electric field for computing the DEP force acting on the cells. The stretching experiment was completed within 30 min in order to minimize the threat to the survival of the cells [37].

### Experimental setup for OT cell stretching

An optical tweezers system (BioRyx 200) that can generate multiple optical traps was utilized to conduct the stretching experiments. Prior to the experiments, streptavidin-coated polystyrene beads with a radius of 1.55 μm were coated with biotin-conjugated concanavalin A at 4 °C for 40 min. The coated beads were then rinsed and incubated with the cells to enable attachment between the beads and the cells.

The cells were loaded on a glass slide and only cells with two beads attached on opposite sides of the diameter line were chosen for the experiments. One optical trap was used to hold one bead in place while one optical trap was used to manipulate the opposing bead until the bead escaped from the trap. The laser power used is 0.5, 1, 1.5, 2, 2.5 and 3 W, respectively, and details of the experiments can be referenced to the work in [9].

### Computation of DEP force

In this work, three different approaches were considered to estimate the DEP force acting on the cells. The first approach employs the widely adopted DEP force equation [39, 40]:

1 $$ F_{DEP} = 2\pi r^{3} \varepsilon_{0} \varepsilon \text{Re} \left[ {K(\omega )} \right]\nabla E^{2} $$

where *r* is the cell radius, which is approximately 7 μm for both NB4 and NB4-DOX cells as measured using ImageJ software; *ε*_*0*_ is a dielectric constant of the vacuum, which is 8.854 × 10^−12^ F/m; *ε* is the relative dielectric constant of the DEP medium, which is 78; *E* is the electric field; and $\nabla$ is the del (gradient) operator; Re[K(ω)] is the real part of the Clausius–Mossotti (CM) factor, which is dependent on the angular frequency (ω) of the applied potential, as well as the dielectric properties of the cell and the medium.

The expression above is based on the equivalent dipole moment (EDM) method used to derive the net force induced at the two poles of a polarized cell. To compute the force, the gradient of the square of an electric field, which is dependent on the geometry of microelectrodes, is required and this can be obtained through computer simulation [20, 41] or analytically using the boundary element method [42].

Alternatively, the DEP force can be calculated by integrating the Maxwell stress tensor (MST) over the surface of the cell to yield the force. For general tip-to-tip electrode configuration, Engelhardt et al. [23] proposed a simple approximation by assuming the electric field inside the cell is small as compared to the field outside, and the force can thus be estimated as [23]:

2 $$ F_{DEP} = \frac{1}{4}\varepsilon_{0} \varepsilon E^{2} A $$

where the electric field is *E* = *U*/*d*, in which *U* is the applied potential and *d* is the electrode gap (20 μm). *A* is the surface area of the cell. This rough approximation also neglects the effect of the applied frequency, which could lead to a change in the DEP force between positive and negative at various frequencies. For a better force estimation, Wang et al. [43] adopted the phasor representation for the electric field (E = E_0_e^iwt^) and the expression becomes [43, 44]:

3 $$ F_{DEP} = \frac{1}{4}\varepsilon_{0} \varepsilon \int\limits_{A} {\left( {EE^{*} + E^{*} E - \left| E \right|^{2} } \right)} \cdot \hat{n}dA $$

where E* is the complex conjugate of the electric field and $$ \overset{\lower0.5em\hbox{$\smash{\scriptscriptstyle\frown}$}}{n} $$ is the unit vector normal to A.

### Actin cytoskeleton modeling

We previously developed an actin microstructural model by using F-actin and ABPs to characterize the mechanical properties of cells [26]. In the model, actin filaments are randomly distributed to form the 3D actin cytoskeleton network and each filament is modeled to exhibit the nature of a semiflexible polymer. The ends of any two filaments are connected randomly by ABPs, which are represented by linear springs. Under cell stretching condition, the force acting on the *i*th vertex (*F*_*i*_) can be consisted of the internal forces of the actin filaments (*f*_*a*_) and the ABPs (*f*_*c*_) connected to the vertex, as well as the external stretching force (*f*_s_). Force balance was derived using the Newton’s equations of motion to determine the positions of all actin vertices; these equations are expressed as follows:

4 $$ m_{i} \ddot{r}_{i} + \eta \dot{r}_{i} = F_{i} $$

5 $$ F_{i} = \sum\limits_{i = 1}^{{n_{a} }} {f_{a\_i} } + \sum\limits_{i = 1}^{{n_{c} }} {f_{c\_i} + f_{s} } $$

where *r*_*i*_ = [*x*_*i*_ *y*_*i*_ *z*_*i*_]^T^ denotes the position of the *i*th vertex of the actin network, *m*_*i*_ is the fictitious mass of the *i*th vertex, *η* is the viscosity of the cytoplasm, *n*_*a*_ is the number of actin filaments, and *n*_c_ is the number of ABPs. Force-extension behavior of ABPs can be represented by linear Hookean springs with a stiffness of *k*_*c*_, and the force-extension behavior of actin filaments can be modeled using MacKintosh-derived WLC model, as follows [45]:

6 $$ f_{a} = \frac{{81k_{b} TL_{p}^{2} L_{c}^{2} (\Delta r + \delta r_{0} )}}{{(L_{c}^{2} - 6L_{p} \Delta r - 6L_{p} \delta r_{0} )^{2} (L_{c}^{2} + 3L_{p} \Delta r + 3L_{p} \delta r_{0} )}} $$

where Δ*r* is the extension of the actin filament, *δr*_*0*_ is the pre-extension of the actin filament caused by prestress, *L*_*p*_ is the persistence length, *L*_*c*_ is the contour length, *k*_*b*_ is the Boltzmann’s constant, and *T* is the absolute temperature. In addition, the relationship among contour length (*L*_*c*_), diameter of F-actin (*d*_*Actin*_), actin concentration (*C*_*AF*_), and density of the crosslinks (*R*) of actin network [45] can be expressed as follows:

7 $$ L_{c} = \frac{{R^{0.2} d_{Actin} }}{2}\sqrt {\frac{\pi }{{C_{AF} }}} $$

Through the model, the influence of parameters such as the actin concentration, density of cross-link, and the prestress effect on the actin cytoskeleton can be quantitatively analyzed.

### Experimental procedure

The structural parameters of the cell model were obtained through fitting of experimental data. NB4 and NB4-DOX cells were stretched under different DEP forces, and deformations were evaluated from the captured images. As discussed in Eq. (1), the strength of the DEP force is dependent on the size of the cell, the applied frequency, and the electric field gradients from the electrodes. The real part of the CM factor is a frequency dependent parameter that is bounded between −0.5 and 1 [40]. A typical biological cell can maintain a CM factor of around 1 in the mega-hertz frequency range. To select an appropriate operating frequency for a voltage input, a frequency range of 100 Hz to 5 MHz was examined to observe the movements of NB4 cells. At the maximum frequency of 5 MHz, the NB4 cells in a low-conductivity medium (5.29 mS/m) can still be manipulated and captured by one of the microelectrodes via positive DEP (pDEP) effect. However, when the frequency was gradually reduced to 25 kHz or lower, NB4 cells started to detach or repel from the microelectrode, indicating a switch from pDEP to negative DEP (nDEP) effect. This work used an operating frequency of 1 MHz, which can provide the maximal CM factor and minimize the Joule heating effect on the cells [24].

**Fig. 3:**
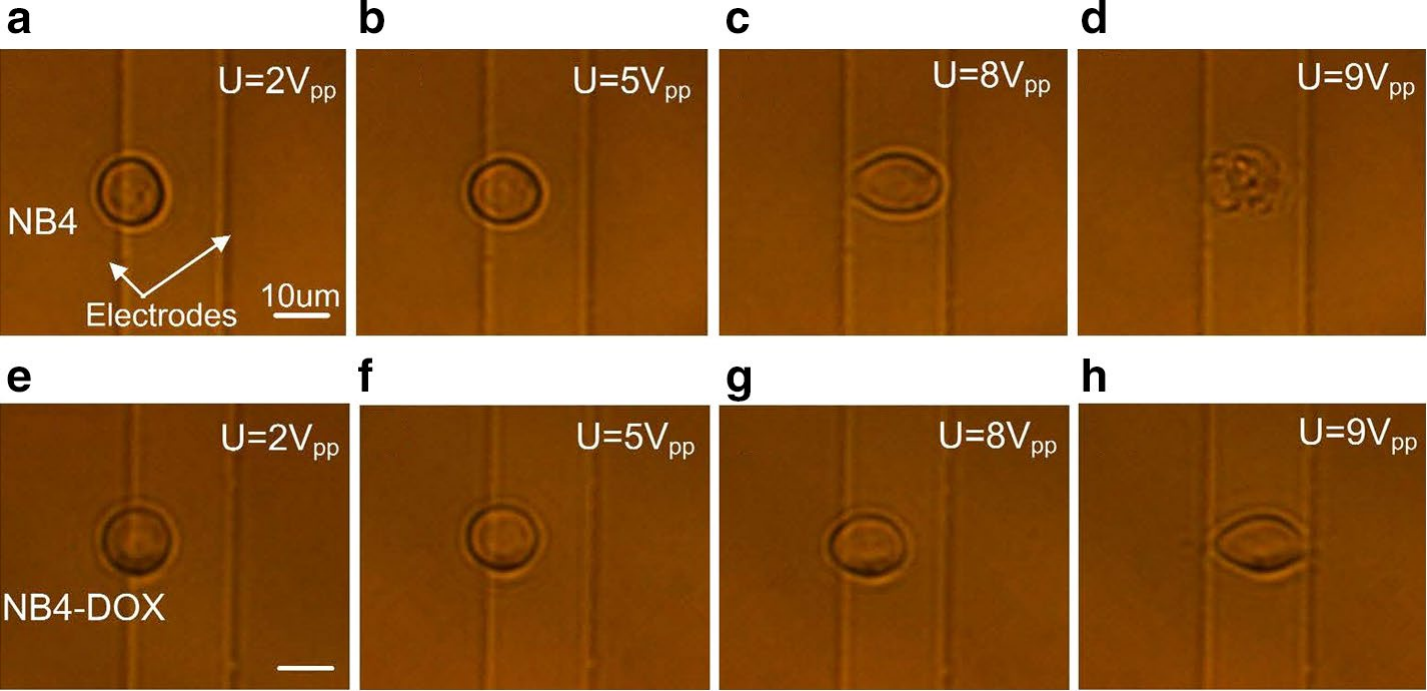
Deformation of an NB4 cell under different voltages: **a** the cell was captured at the electrode edge using 2 V_pp_ and then deformed under **b** 5 V_pp_, **c** 8 V_pp_; **d** the cell lysed under 9 V_pp_. Deformation of an NB4-DOX cell: **e** the cell was captured at the electrode edge using 2 V_pp_ and then deformed under **f** 5 V_pp_, **g** 8 V_pp_, and **h** 9 V_pp_

The NB4 cells were stretched to different scales by adjusting the strength of the electric field. An initial sinusoidal voltage input of 2 V_pp_ (peak-to-peak) was first applied to capture and immobilize the NB4 cells, and then the voltage amplitude was adjusted to 3, 4, 5, 6, 7, 8, and 9 V_pp_ to stretch the NB4 cells for 3 min. The same stretching experiments were performed for NB4-DOX cells. Figure 3a–d show the deformation of an NB4 cell, and Fig. 3e–h show the deformation of an NB4-DOX cell under different voltages. The deformation along the long axis of the ellipsoidal cell was measured using ImageJ software [19, 41].

## Results and discussion

### Numerical simulation

**Fig. 4:**
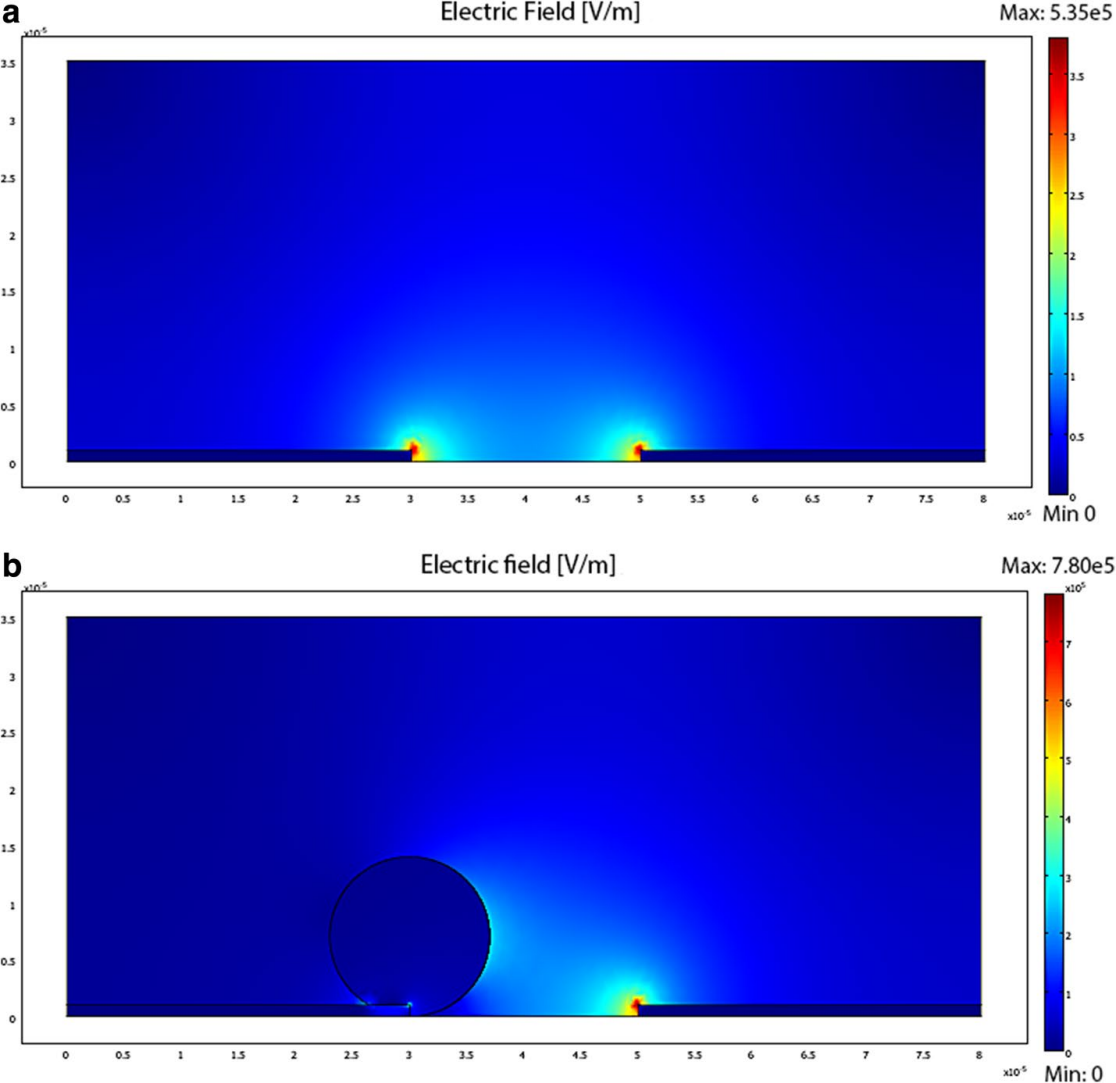
COMSOL simulation showing the electric field distribution **a** without and **b** with the cell

The DEP forces generated under different voltage inputs were calculated using the three approaches described in “Methods”, and the results are summarized in Table 1. The simplified force equation proposed by Engelhardt et al. [23] was directly utilized by substitution using the experimental values of the parameters. To estimate the gradient terms in the EDM equation, we simulated the electric fields generated the microelectrodes using COMSOL (Fig. 4a), and the simulation setup was similar to those in [41, 46]. Briefly, we modeled the 2D cross-sectional view of the microfluidic chip and exported the electric field strengths near the center of the cell to compute the field gradients [41]. To evaluate the stress tensor, we added a dielectric particle with a radius of 7 μm into the model, and we integrated the MST acting on the particle surface to obtain the DEP forces (Fig. 4b). The governing Maxwell’s equation selected to be solved is in the transient form:

8 $$ - \nabla ((\sigma + j\omega \varepsilon_{0} \varepsilon )\nabla \phi ) = 0 $$

with the electric field, E, and the displacement, D, are defined as:

9 $$ E = - \nabla \phi $$

10 $$ D = \varepsilon_{0} \varepsilon E $$

**Table 1:** Estimation of the DEP forces by using different approaches

Voltage inputs (V)	DEP force (nN)		
	EDM	MST	Simplified
1.5	0.16	0.47	0.61
2	0.28	0.72	1.09
2.5	0.44	1.13	1.70
3	0.64	1.63	2.45
3.5	0.86	2.23	3.34
4	1.14	2.91	4.36

In the COMSOL model, conditions for the boundaries between the cells and medium were set as continuous and boundaries of the microfluidic chip were set as electrically insulated. One electrode was set as the applied potential and the other electrode was set as the ground [18, 19, 47]. The simulated electric fields were multiplied with the normal vectors and integrated over the cell boundary to obtain the DEP force as [44]:

11 $$ \left[ {\begin{array}{*{20}c} {F_{DEP,x} } \\ {F_{DEP,y} } \\ \end{array} } \right] = \frac{1}{4}\varepsilon_{0} \varepsilon \int\limits_{A} {\left[ {\begin{array}{*{20}l} {E_{x} E_{x}^{*} + E_{x}^{*} E_{x} - (E_{x} E_{x}^{*} + E_{y} E_{y}^{*} )} \hfill & {E_{x} E_{y}^{*} + E_{x}^{*} E_{y} } \hfill \\ {E_{y} E_{x}^{*} + E_{y}^{*} E_{x} } \hfill & {E_{y} E_{y}^{*} + E_{y}^{*} E_{y} - (E_{x} E_{x}^{*} + E_{y} E_{y}^{*} )} \hfill \\ \end{array} } \right]} \cdot \hat{n}dA $$

### Comparison of the approaches for DEP force estimation

Comparison of the results shows that the forces estimated from the three approaches all lie within the nanoNewton range, indicating that each approach demonstrates good approximations. Among the three approaches, the simplified approach can provide a quick estimation on the force, although the value could be overestimated. The EDM approach yields a lower force estimation than the MST approach, which is consistent with the findings in [48]. The reason could be due to the approximation in the EDM approach, which neglects higher order terms in the computation. In addition, the length of the dipole (cell) is assumed to be smaller than the non-uniformity of the electric field so that the electric field does not vary significantly across the dipole [40]. The MST approach, generally, should provide the most accurate result and alterations in electric field distributions caused by the presence of a dielectric particle of any size can be accounted for in the computer simulation.

Since both the EDM and the MST approaches rely on the data of the electric field generated from the simulation, approximations or assumptions in the computer model could affect the accuracy of the computed DEP force as compared to the actual DEP force. Similar to the work in [19, 41, 47], a 2D model was considered to reduce the computational burden. A side-view, rather than a top-view model of the microfluidic chip was adopted in this work because it can better model the configuration of the current experimental setup. The thickness of the electrodes is much thinner than the cell, and a top-view model could lead to a significant overestimation on the electric field acting on the cell surface along the out-of-plane direction. Nevertheless, in the side-view model, the geometry of the electrodes in the sidewall is smaller than the cell and the result could be more sensitive to the mesh size used in the simulation. In the MST approach, the 2D tensors along the cell surface were integral and multiplied by a constant (thickness in the out-of-plane direction) to obtain the DEP force in 3D. This geometrical approximation could also lead to an error in estimating the DEP force acting on the cell.

### Comparison between DEP and OT stretching

**Fig. 5:**
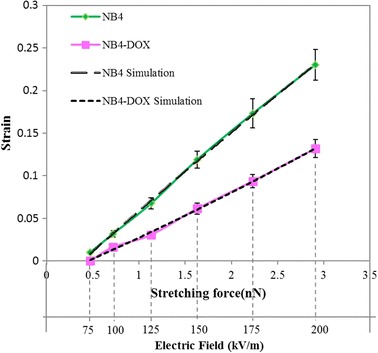
Strain–force curves of NB4 and NB4-DOX cells under DEP stretching (mean ± SE, NB4 cells: n = 54, NB4-DOX cells: n = 55)

**Fig. 6:**
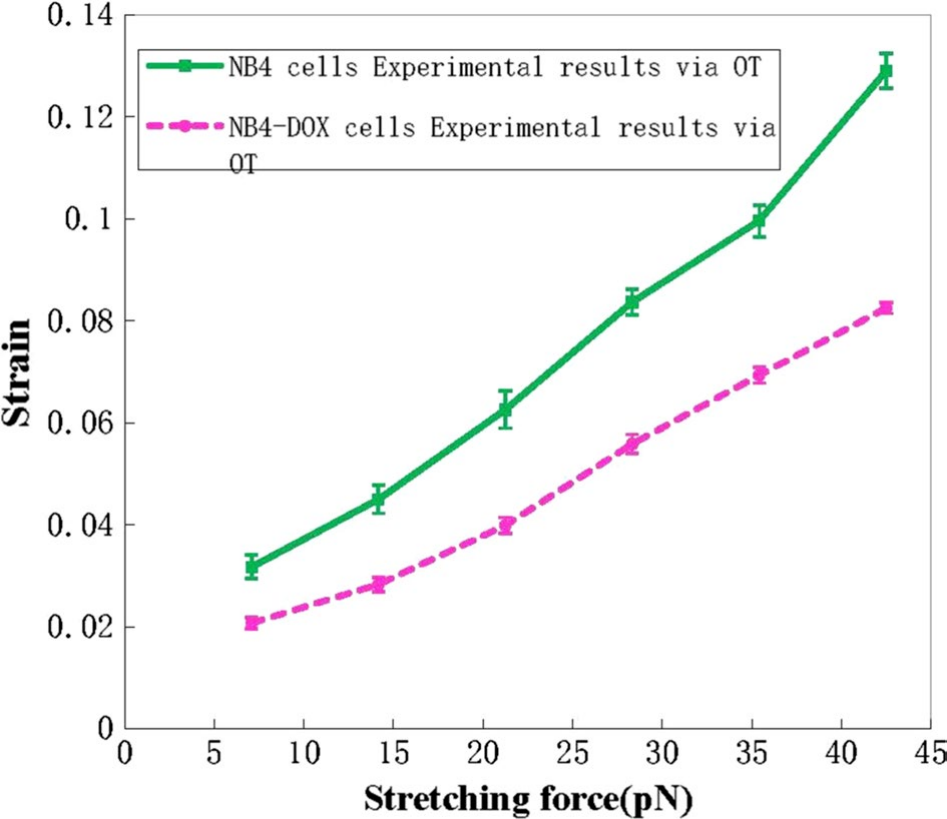
Strain–force curves of NB4 and NB4-DOX cells under optical tweezer (OT)-based stretching (mean ± SE, NB4 cells: n = 20, NB4-DOX cells: n = 20)

The performance of the DEP stretching method was compared with that of the standard OT stretching method. First, NB4 and NB4-DOX cells were stretched using DEP with different voltage inputs, and their deformations were measured using an optical microscope. The corresponding DEP forces were computed using the MST approach, and the results are plotted in Fig. 5. The strain of the deformed cell was calculated using (*r*–*r*_0_)/*r*_*0*_, where *r* and *r*_*0*_ denote the current and initial radii of the cell, respectively. In order to account for slight variation on the cells, more than 50 cells (n > 50) were examined for each group. Experimental results show that NB4 cells were stiffened after DOX treatment. Under a 2.9 nN force input, the average strain of NB4 cells is 0.23, whereas the average strain of NB4-DOX cells is 0.13. Other sets of NB4 and NB4-DOX cells were used in OT stretching, and the results are plotted in Fig. 6. Under a 43 pN force input, the average strains in NB4 and NB4-DOX cells are 0.13 and 0.08, respectively.

The elastic moduli for NB4 and NB4-DOX cells were estimated by relating the stress (Force/area) to the deformation using the method described in [47]. Based on the experimental data, the moduli for NB4 and NB4-DOX cells from the DEP results were approximately 36 and 62 Pa, respectively, while the moduli from the optical tweezers results were only 1.2 and 1.8 Pa, respectively. The R^2^ value obtained from the two curves were >0.98, indicating good fit for the data. Similar to the findings reported in [22, 47], the DEP method was found to yield a higher force value as compared to other characterization methods such as AFM.

The results obtained from the two methods may not be used for direct comparison. Although similar cell deformation behaviors were observed in both methods after DOX treatment, the range of the applied stretching force differs by two orders of magnitude mainly because of the intrinsic characteristics of each stretching mechanism. Several research groups have attempted to compare the mechanical properties of cells using various methods, and significant discrepancies were also reported. For instance, Laurent et al. [49] compared the elastic modulus of epithelial cells measured using magnetic cytometry and OT, and a discrepancy of up to fivefold was observed. They concluded that bead attachment could strongly affect the force applied to the cells, and increased stress in magnetic cytometry could reinforce nonlinearity and cellular plasticity. Urbano et al. [22] examined attached epithelial cells by using DEP pushing and AFM indentation method and found that the elastic modulus differed by at least 10 times. Fortier et al. [50] used AFM to characterize leukemia cells with two different probes and they found that the elastic moduli evaluated from a conical probe were larger, and their distribution was wider, as compared to the results from a spherical probe.

The loading rate corresponded to the stretching mechanism is another factor that has an influence on the deformability of the cell. In the OT method, the cell is gradually stretched by the bead in order to prevent the bead from escaping the optical trap during stretching. In contrast, the cell in the DEP method is directly stretched by the electric field supplied by the function generator, and any adjustment in the supply voltage will immediately apply onto the cell. As reported in [51], the loading rate and the apparent elastic modulus are correlated and a higher loading rate can lead to a higher apparent elastic modulus. Hence, for the same deformation, it is expected that the DEP method should require a higher stretching force. Other research groups have also been examined the cell behavior with different loading rates. Pravincumar et al. [52] reported that slow rates of applied pressure or deformation could result in high level of cortical actin distortion, affecting the mechanical properties or elastic modulus of a cell to be measured. Nawaz et al. [53] indicated that the elastic modulus of 3T3 cells increased from 140 Pa at a low deformation rate to 330 Pa at a high deformation rate. Hence, slight variations in factors such as magnitude of the force applied, contact area, and deformation speed could lead to a wide range of cell deformations in various methods.

The effect of the electric field on NB4 cells could be another factor causing discrepancies in the experimental results. As reported by Wigge et al. [54] and Titushikin et al. [55], the use of electric field can alter the alignment of the actin filament in the cytoskeleton structure. When biological cells were exposed to the electric field for 60 min, the elasticity of hMSC cells was reported to decrease by 70% while the elasticity of osteoblast cells only dropped by 30%. The decrease in the cell elasticity could be related to the increase in the cell stiffness as the cell progresses towards cell death [56].

### Probing the biomechanical properties of NB4 cells using DEP-based stretching combined with actin cytoskeleton modeling

The structural parameters in the model were evaluated by using the experimental data with the iterative approach as described in [26] and the obtained values for different cytoskeleton parameters are summarized in Table 2. The results revealed that the internal (*R*_*I*_) radius, external (*R*_*E*_) radius, number of F-actin (*N*_*AF*_), and F-actin density (*C*_*AF*_) of the actin network all increased after DOX treatment. In particular, the increase in *C*_*AF*_ can help explain the DOX-induced cell-stiffening behavior, where *C*_*AF*_ is strongly correlated to the elastic modulus of a cell [29]. Several studies have also confirmed that a low DOX concentration can cause variation in the dielectric properties of cells, inducing changes in their actin cytoskeleton structure [57, 58].

**Table 2:** Estimation of the structural parameters of the actin cytoskeleton of NB4 and NB4-DOX cells; these parameters were extracted through cell modeling and stretching manipulation using DEP

Cell type	*R*_*E*_ (μm)	*R*_*I*_ (μm)	*C*_*AF*_ (μM)	*N*_*AF*_
NB4	7.02	6.21	152	53,531
NB4-DOX	7.32	6.22	325	162,962

*R*_*E*_ is the external radius of actin network

*R*_*I*_ is the internal radius of actin network

*C*_*AF*_ denotes F-actin density

*N*_*AF*_ is the number of F-actin in actin network

**Fig. 7:**
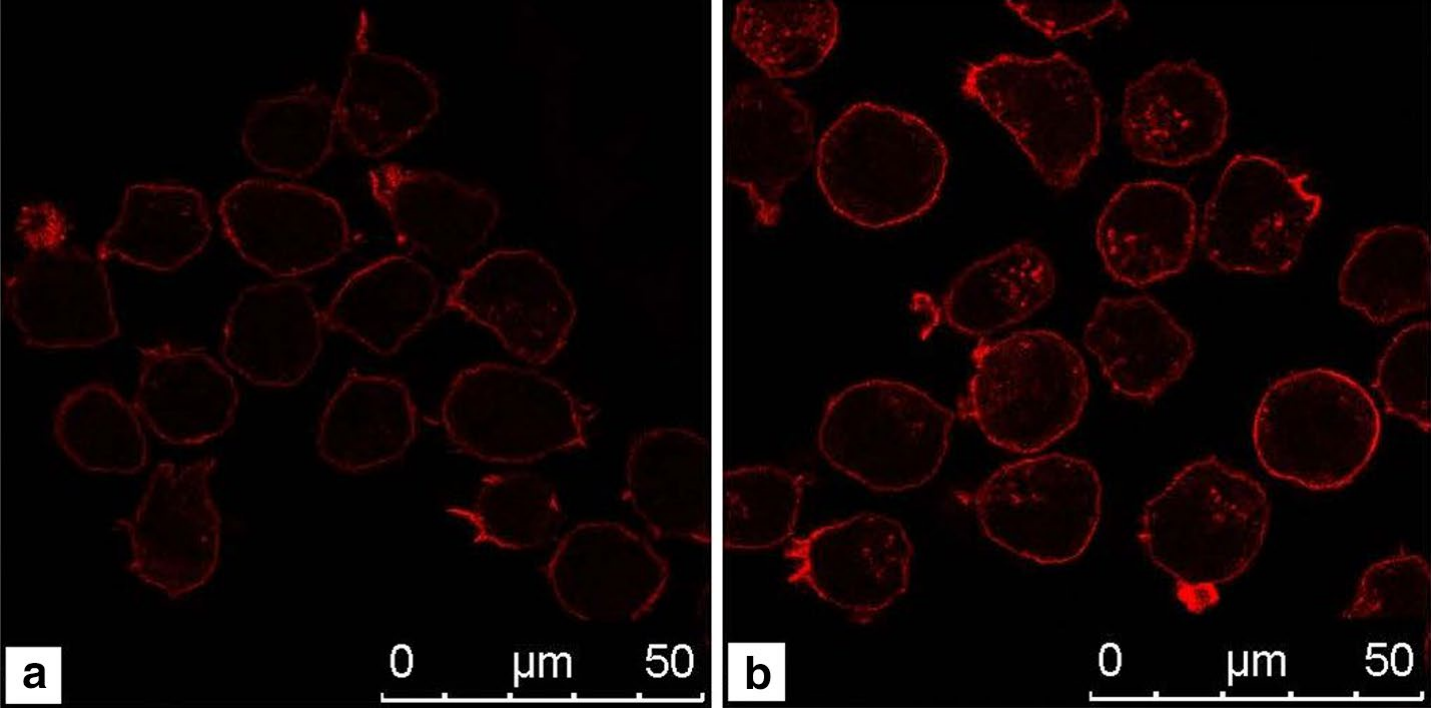
Confocal images of actin cytoskeleton. **a** NB4 cells and **b** NB4-DOX cells (the *scale bar* is 10 μm)

Furthermore, the effects of DOX treatment on NB4 cells were examined through rhodamine-phalloidin staining. Cells were first fixed with 4% paraformaldehyde and then permeabilized with 0.5% Triton X-100 for 10 min. Cells were treated with blocking buffer (10% BSA in PBS) for 20 min and the actin cytoskeleton was stained with rhodamine-phalloidin for 10 min at room temperature [9]. Confocal images (Fig. 7) showed that after drug treatment, NB4-DOX cells display an increased density of F-actin, which was distributed within a significantly large area. This result is in consistent with the findings in Table 2. This result demonstrates DOX-induced actin cytoskeleton remodeling, along with formation of a cortical contractile ring at the cell periphery [59].

## Conclusions

This paper presents a new method to probe the mechanical properties of cells through DEP-based cell stretching manipulation and actin cytoskeleton modeling. In this work, NB4 cells and NB4 cells treated with drug were examined and results from DEP cell-stretching showed that the deformability of NB4 cells decreased after DOX treatment. Similar results were also obtained using the OT-based stretching method. Through experimental data, structural parameters in the model can be extracted so that the correlation between the actin cytoskeleton structure and the cell mechanical properties can be quantitatively characterized. The results demonstrated that the proposed DEP method combined with actin cytoskeleton modeling can provide a simple and cost-effective engineering tool to characterize the mechanical properties of cells; moreover, this method can serve as an important alternative to the OT-based stretching method.

## Abbreviations

ABP: actin-binding protein
DEP: dielectrophoresis
DOX: doxorubicin
OT: optical tweezers
WLC: worm-like chain
